# Identification of nucleotides and amino acids that mediate the interaction between ribosomal protein L30 and the SECIS element

**DOI:** 10.1186/1471-2199-14-12

**Published:** 2013-06-19

**Authors:** Abby L Bifano, Tarik Atassi, Tracey Ferrara, Donna M Driscoll

**Affiliations:** 1 Department of Cellular & Molecular Medicine, Lerner Research Institute, Cleveland Clinic, 9500 Euclid Avenue NC10, Cleveland, OH 44195, USA; 2 Present address: Department of Human Medical Genetics, University of Colorado, Aurora, CO, USA; 3 Department of Molecular Medicine, Cleveland Clinic Lerner College of Medicine of Case Western Reserve University, Cleveland, OH, USA

**Keywords:** RNA-binding protein, L30, SECIS-binding protein 2, Selenocysteine, Selenoprotein

## Abstract

**Background:**

Ribosomal protein L30 belongs to the L7Ae family of RNA-binding proteins, which recognize diverse targets. L30 binds to kink-turn motifs in the 28S ribosomal RNA, L30 pre-mRNA, and mature L30 mRNA. L30 has a noncanonical function as a component of the UGA recoding machinery that incorporates selenocysteine (Sec) into selenoproteins during translation. L30 binds to a putative kink-turn motif in the Sec Insertion Sequence (SECIS) element in the 3’ UTR of mammalian selenoprotein mRNAs. The SECIS also interacts with SECIS-binding protein 2 (SBP2), an essential factor for Sec incorporation. Previous studies showed that L30 and SBP2 compete for binding to the SECIS in vitro. The SBP2:SECIS interaction has been characterized but much less is known about how L30 recognizes the SECIS.

**Results:**

Here we use enzymatic RNA footprinting to define the L30 binding site on the SECIS. Like SBP2, L30 protects nucleotides in the 5’ side of the internal loop, the 5’ side of the lower helix, and the SECIS core, including the GA tandem base pairs that are predicted to form a kink-turn. However, L30 has additional determinants for binding as it also protects nucleotides in the 3’ side of the internal loop, which are not protected by SBP2. In support of the competitive binding model, we found that purified L30 repressed UGA recoding in an in vitro translation system, and that this inhibition was rescued by SBP2. To define the amino acid requirements for SECIS-binding, site-specific mutations in L30 were generated based on published structural studies of this protein in a complex with its canonical target, the L30 pre-mRNA. We identified point mutations that selectively inhibited binding of L30 to the SECIS, to the L30 pre-mRNA, or both RNAs, suggesting that there are subtle differences in how L30 interacts with the two targets.

**Conclusions:**

This study establishes that L30 and SBP2 bind to overlapping but non-identical sites on the SECIS. The amino acid requirements for the interaction of L30 with the SECIS differ from those that mediate binding to the L30 pre-mRNA. Our results provide insight into how L7Ae family members recognize their cognate RNAs.

## Background

Eukaryotic ribosomal protein L30 is a component of the large ribosomal subunit. L30 has no prokaryotic ortholog but the gene is essential in yeast [1]. Cryo-electron microscopy studies of the wheat germ and canine 80S ribosomes revealed that L30 is located in a eukaryotic-specific bridge between the large and small subunits [2,3]. The interaction of L30 with the 60S ribosome is mediated primarily through binding of the protein to a kink-turn motif in helix 58 of the large rRNA [2,4]. L30 also binds to a kink-turn in the 5’ untranslated region (UTR) of its cognate pre-mRNA and the mature spliced mRNA to auto-regulate its own expression at the level of pre-mRNA splicing or mRNA translation, respectively [5-8].

**Figure 1:**
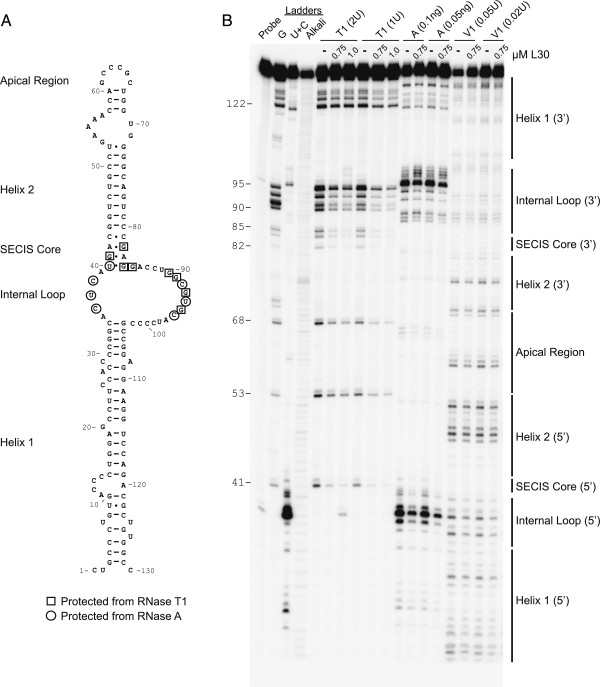
**RNA footprinting of the L30:SECIS complex. A**. The structure of the SECIS element from the rat PHGPx mRNA is shown. Boxes and circles indicate nucleotides that are protected from cleavage by RNase T1 and RNase A, respectively. **B**. The 5’ end-labeled PHGPx SECIS was incubated in the absence or presence of L30 (0.75 or 1.5 μM). The reactions were then partially digested with RNase T1, A, or V1 as indicated. The products were analyzed by denaturing gel electrophoresis. The sequencing (G and C + U) and alkali ladders are shown in the left lanes. The numbers to the left of the gel indicate the positions of G nucleotides using the numbering in (A). The bars on the right indicate the different regions of the SECIS element. The gel is a representative example from 3 independent experiments.

The repertoire of L30 functions was expanded by the discovery that the protein is involved in the mechanism that recodes the UGA stop codon as selenocysteine (Sec) during selenoprotein synthesis [9]. In humans, there are 25 known selenoprotein genes, whose products play critical roles in anti-oxidant defense, thyroid hormone metabolism, immunity, and development [10,11]. Sec incorporation at the UGA/Sec codon is dependent on a stem-loop structure, the Selenocysteine Insertion Sequence (SECIS) element, which is found in the 3’ UTR of eukaryotic selenoprotein mRNAs. This structure consists of two helices separated by an internal loop with an apical loop or bulge at the top (see Figure 1A). The core of the SECIS contains a quartet of non-Watson-Crick base pairs, including two sheared G•A tandem base pairs which are characteristic of kink-turn motifs. Based on structure probing and computer modeling, Walczak et al. proposed a three-dimensional structure of the SECIS in which the RNA is kinked at the internal loop, exposing the sheared G•A tandem base pairs in the SECIS core to the solvent [12]. The SECIS core, which is essential for Sec incorporation, is required for binding of two proteins, L30 and SECIS-binding protein 2 (SBP2) [9,13-16]. *In vitro* studies support a model in which the two proteins bind to the SECIS element independently, and likely sequentially [9]. SBP2 has been shown to recruit the Sec-tRNA^Sec^:EFSec complex, bind to the ribosome, and induce a conformational change in the A site [17-19]. However, the exact functions of SBP2 and L30 in UGA recoding have not been fully defined, and alternative models have been proposed [9,17,18].

A prerequisite for elucidating the mechanism of Sec incorporation is a detailed understanding of the molecular basis for protein:SECIS interactions. L30 and SBP2 are both members of the L7Ae family of RNA-binding proteins. In addition to the founding member archaeal ribosomal protein L7Ae, this family includes other eukaryotic ribosomal proteins as well as proteins involved in RNA processing, ribonucleoprotein assembly, and termination of protein synthesis [20,21]. The L7Ae family members share a similar RNA-binding domain and characteristically bind to kink-turn motifs in their cognate RNA. However, the kink-turn does not represent a single structural motif [22]. Each protein in the L7Ae family has a unique RNA-binding specificity, which allows it to distinguish its cognate RNA from other kink-turn containing transcripts in the cell. Indirect evidence from our lab suggests that the SECIS core is part of a noncanonical kink-turn, which may explain why the SECIS is bound by L30 and SBP2, but not by other proteins in the L7Ae family [9].

We, and others have characterized the SBP2:SECIS interaction at the molecular level, although no structural studies on the complex have been performed to date. Based on RNA footprinting experiments, SBP2 binds to both sides of the SECIS core, as well as to the 5’ strand of the internal loop and lower helix [14]. The upper helix and a large internal loop in the SECIS may also be important for recognition by SBP2 [23]. Mutational analysis of SBP2 revealed that the L7Ae motif is necessary but not sufficient to mediate SECIS-binding and that additional amino acids in a K-rich region N-terminal to this motif are required [24,25]. The SBP2:SECIS interaction is critical for human health as mutations in either the SBP2 binding site or in the SBP2 RNA-binding domain result in a reduction in selenoprotein synthesis and a variety of phenotypes [26-28].

In contrast to SBP2, much less is known about how L30 recognizes the SECIS. We previously showed that L30 binds to SECIS elements from multiple selenoprotein mRNAs [9], but the actual L30 binding site has not been defined. Compared to SBP2, L30 is a relatively small protein (11 kDa) and it lacks a K-rich region. Structural studies of L30 in a complex with its cognate target, the stem-loop from the L30 pre-mRNA, have defined the RNA-binding interface of the protein [29,30]. However, it is not known whether the same amino acids mediate binding to the SECIS. In this study, we used RNA footprinting and site-directed mutagenesis to identify nucleotides and amino acids that are important for the L30:SECIS interaction.

## Results

### Defining the L30 binding site by RNA footprinting

Eukaryotic SECIS elements are stem-loop structures containing two highly conserved motifs that are essential for Sec incorporation, namely the SECIS core and the AAR motif in an apical bulge or loop. The SECIS element from the rat Phospholipid Hydroperoxide Glutathione Peroxidase (PHGPx) mRNA is shown in Figure 1A. We previously showed that binding of L30 to the PHGPx SECIS was abrogated by mutations in the sheared G•A tandem base pairs in the SECIS core, but not by deletion of the AAR motif [9]. In order to define the L30 binding site on the SECIS, we used enzymatic RNA footprinting. The ^32^P-labeled PHGPx SECIS RNA was incubated in the presence or absence of purified rat L30. The native RNA and RNA:protein complexes were then partially digested with different ribonucleases and analyzed by electrophoresis. Regions of cleavage and protection were identified by comparing samples with RNA sequencing reactions (G and C + U) and alkali ladders. A schematic illustrating the results is shown in Figure 1A and a gel representative of 3 independent experiments is shown in Figure 1B.

The cleavage results with the native RNA are consistent with the published structure of the PHGPx SECIS, which was determined by enzymatic and chemical probing [31]. RNase T1, which cleaves after single-stranded G bases, cleaved the native PHGPx SECIS RNA in the apical region, both strands of the SECIS core, and the 3’ side of the internal loop (Figure 1B). Similar results were obtained when the SECIS was partially digested with RNase A, which cleaves at single-stranded C and U bases. Cleavage by RNase A was detected in the 5’ side of the SECIS core, the apical loop, as well as both sides of the internal loop. Both RNase A and T1 cleaved at the base of helix 1 (nucleotides 121-128) suggesting that this region may breathe due to imperfect base pairing. RNase V1, which cleaves in double-stranded regions, cleaved at multiple positions in helix 1 and helix 2. We also observed faint V1 digestion in the large internal loop, which suggests that this region may occasionally form an alternate structure.

When the SECIS was incubated with 0.75 or 1.5 μM L30, there was a marked reduction in cleavage by RNase T1 on both sides of the SECIS core (G41, G82, G84) and along the 3’ side of the internal loop 2 (G85, G90, G91, G93, G95). We were unable to achieve full protection by increasing the amount of L30 in the binding reaction, most likely due to the high on/off rate of the L30:SECIS complex as determined by Surface Plasmon Resonance (data not shown). G53 and G68 in the apical region and G122, G125, G127, and G128 at the base of helix 1 were not reproducibly protected by L30 from RNase T1 cleavage.

**Figure 2:**
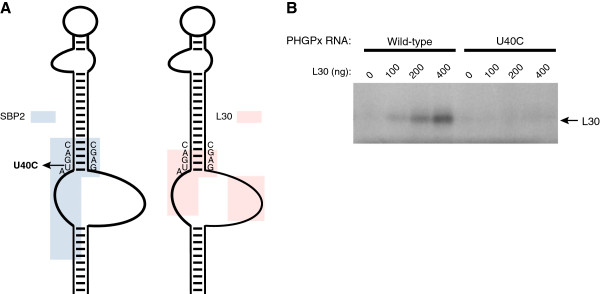
**The U40C mutation abrogates L30 binding. A**. Schematic illustrating the binding sites of SBP2 [14] and L30 (this work) on the SECIS, as determined by RNA footprinting. The position of the U40C point mutation is indicated. **B**. UV cross-linking experiments were performed using the ^32^P-labeled wild-type PHGPx SECIS or the U40C mutant RNA, which were incubated with increasing amounts of purified L30 as indicated. After RNase digestion, the products were analyzed by SDS-PAGE and autoradiography.

When the L30:SECIS complexes were partially digested with RNase A, there was a reduction in cleavage in the 5’ side of the internal loop and SECIS core (bases 36-38 and 40) and the 3’ side of the internal loop (bases 92, 94, 96). Other bases in the internal loop (C87, C88, U89) and the base of helix 1 were still cleaved by RNase A in the presence of L30. Similarly, the binding of L30 did not protect nucleotides in helix 1 or helix 2 from RNase V1 cleavage. Taken together, our results show that binding of L30 protects nucleotides in the SECIS core and in the 5’ and 3’ sides of the internal loop. We previously showed that SBP2 interacts with both sides of the SECIS core, the 5’ strand of the internal loop, and the 5’ strand of helix 1, but not with the 3’ side of the internal loop [14]. Thus the two proteins bind to similar but not identical regions of the SECIS, as illustrated in Figure 2A.

### L30 represses UGA recoding in vitro and this inhibition is rescued by SBP2

The fact that the SBP2 and L30 binding sites overlap is consistent with our earlier finding that purified recombinant L30 and SBP2 compete for binding in vitro to an isolated SECIS in the absence of other factors [9]. To test whether this competition could occur during translation, we used a luciferase reporter construct and a modified rabbit reticulocyte lysate (RRL) system. This assay has been previously validated to be specific for UGA recoding [32]. The Luc/UGA/PHGPx reporter RNA contains a UGA/Sec codon at position 258 in the open reading frame and the PHGPx SECIS in the 3’ UTR. Since RRL contains very little SBP2, we added recombinant SBP2-CT, which represents the C-terminal half of the protein and encodes all known functions of SBP2. Translation assays were supplemented with purified 70 nM SBP2-CT to be within the linear range of the assay. Reactions were performed in the absence or presence of increasing amounts of L30, and the products were analyzed for luciferase activity.

**Figure 3:**
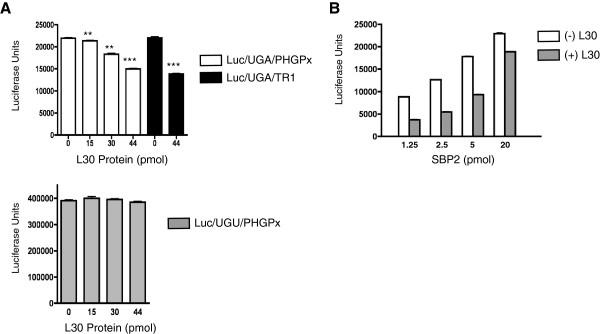
**Repression of UGA recoding by L30 is rescued by SBP2. A**. In vitro UGA recoding assays were performed using a luciferase reporter RNA that contains UGA (top panel) or UGU (bottom panel) at position 258 of the coding region, fused to either the PHGPx or TR1 SECIS element as indicated. Translation assays were performed in the presence of increasing amounts of L30 as indicated. The products were analyzed for luciferase activity using a luminometer, and the results are expressed as means ± SEM. Statistical significance is indicated by ** (*p* < 0.01) and *** (*p* < 0.001). **B**. UGA recoding assays with the luc/UGA/TR1 reporter construct were performed with a constant amount of L30 (44 pmol/reaction) and increasing amounts of SBP2 as indicated. The products were analyzed as described in (A).

As shown in Figure 3A (top panel), the addition of exogenous L30 inhibited recoding of Luc/UGA/PHGPx in a dose-dependent manner. This effect is codon-specific and SECIS-dependent as the inhibition was not observed when the reactions were primed with a Luc/UGU/PHGPx reporter construct, which contains a UGU/Cys codon (Figure 3A, bottom panel) or a normal luciferase RNA that lacks a SECIS element (data not shown). The addition of L30 also repressed UGA recoding directed by the SECIS element from Thioredoxin Reductase 1 (TR1) (Figure 3A, top panel). We hypothesized that the exogenous L30 protein interacted with the SECIS and prevented binding of SBP2, which is limiting in our translation system. To test this competitive model, we added increasing amounts of SBP2 to the translation reaction while keeping the amount of L30 constant. As shown in Figure 3B, the inhibitory effect of L30 on recoding from the TR1 SECIS was rescued by the addition of SBP2. Similar results were obtained using the Luc/UGA/PHGPx construct (data not shown). Taken together, these results demonstrate that SBP2 and L30 can functionally compete in an in vitro translation system.

### Mutations in L30 inhibit SECIS-binding

**Figure 4:**
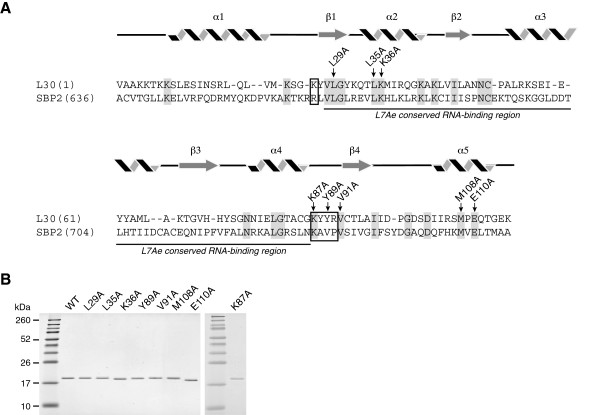
**Mutational analysis of L30. A**. A schematic illustrating the primary sequences and predicted secondary structures of L30 and SBP2. The position numbers refer to the rat protein sequences. The L7Ae conserved RNA-binding domain is underlined and the conserved signature amino acid motifs for L30 and SBP2 are boxed as described in [37]. Arrows indicate the amino acids in L30 that were mutated to alanine. **B**. The wild-type and mutant L30 protein were expressed in bacteria, purified and analyzed by SDS-PAGE and Coomassie Blue staining. The molecular weight markers are shown in the left lane of each gel.

The L7Ae family members are functionally diverse and recognize a variety of targets. A number of these RNA:protein complexes have been analyzed at the structural level [29,30,33-36]. An emerging theme from these studies is that the L7Ae family members bind to their different cognate RNAs in a similar manner. Therefore, we designed site-directed mutations based on the solution structures of yeast L30 in complex with the stem-loop from the L30 pre-mRNA target, which were solved by NMR spectroscopy and x-ray crystallography [29,30]. An induced fit model was proposed in which L30 folds into an α/β/α sandwich, with the three loops at the end of the sandwich in direct contact with the RNA [29,30]. We generated alanine point mutations in these functionally important regions, including L29 in the first loop of the α/β/α sandwich, L35 and K36 in the α2 region, and K87, Y89, and V91 in the α4–β4 loop (Figure 4A). We also mutated M108 and E110 in the C-terminal region, as these residues are present in L30 sequences from higher eukaryotes and SBP2, but not in S12, an L7Ae family member that does not bind the SECIS [9].

**Figure 5:**
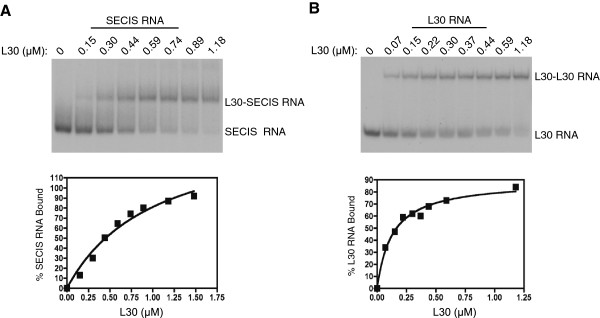
**REMSA analysis of the RNA-binding activity of L30. A**. The ^32^P-labeled PHGPx SECIS was incubated with increasing amounts of L30 as indicated. The complexes were analyzed by native gel electrophoresis (top panel). The percent SECIS bound at each protein concentration is shown graphically (bottom panel). **B**. REMSA analysis was performed as described in (A) except that the stem-loop from the L30 pre-mRNA (L30 RNA) was used as the probe.

**Figure 6:**
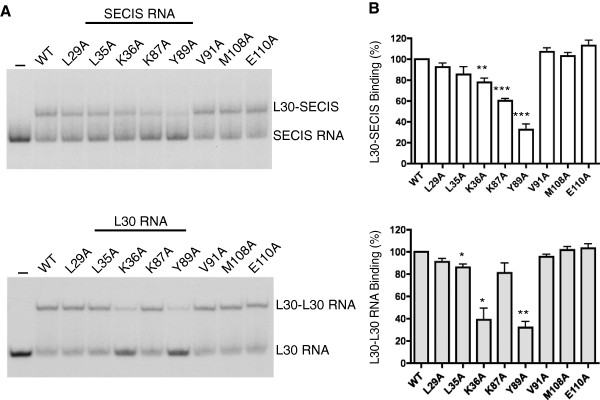
**Point mutations in L30 affect RNA-binding activity. A**. Representative REMSA analysis of the ^32^P-labeled PHGPx SECIS (top panel) or L30 RNA (bottom panel). The RNA probes were incubated in the absence or presence of wild-type and mutant L30 proteins as indicated. The positions of the free probes and protein:RNA complexes are indicated. **B**. Graphical representation of REMSA results for L30 binding to the SECIS (top panel) or L30 RNA (bottom panel) from 5 or 3 independent experiments, respectively. The results are expressed relative to the activity of the wild-type protein, which is expressed as 100%. Statistical significance is shown by asterisks, with * (p < 0.05), ** (*p* < 0.01) and *** (*p* < 0.001).

The wild-type and mutant L30 proteins were analyzed for purity by SDS-PAGE (Figure 4B) and for RNA-binding activity using RNA Electrophoretic Mobility Shift Assays (REMSA). The rat PHGPx SECIS element and the stem-loop structure from the yeast L30 pre-mRNA (subsequently referred to as L30 RNA) were used as ^32^P-labeled probes. As shown in Figure 5A, the wild-type protein bound to the SECIS in a dose-dependent manner with an apparent *K*_D_ of ~ 0.49 μM, which is comparable to what was previously reported [9]. The affinity of L30 for the L30 RNA was several-fold higher with a *K*_D_ of 0.17 μM (Figure 5B). We performed a survey of the mutant proteins, using a protein concentration at which 50% of the probe was bound by the wild-type L30. Representative REMSAs are shown in Figure 6A, and the graphs of the results from 5 and 3 independent experiments, respectively, are presented in Figure 6B.

We found that the amino acid requirements for L30 binding to the SECIS and the L30 RNA are similar but not identical. The Y89A mutant protein was the most defective, with 32% and 37% binding to the SECIS and L30 RNA, respectively, compared to the wild-type protein. We also identified two mutations that selectively inhibited binding of L30 to one target RNA but not the other. As shown in Figure 6B, the K87 mutation in rat L30 reduced binding to the SECIS by 40% but had little effect on the L30:L30 RNA interaction. In contrast the K36A mutant protein was more impaired in its ability to interact with the L30 RNA (reduced by 60%) than the SECIS (reduced by 22%). There was a slight (14%) reduction in binding of the L35A mutant to both the SECIS and L30 RNA, but only the latter result was statistically significant (Figure 6B). The other mutants, L29A, V91A, M108A, and E110A, were comparable to the wild-type protein with respect to their ability to bind to both targets.

**Figure 7:**
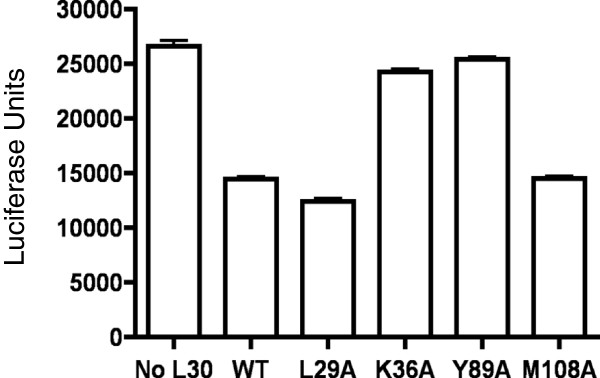
**L30 repression of UGA recoding correlates with SECIS-binding.** UGA recoding assays were performed using the luc/UGA/TR1 reporter construct as described in the legend to Figure 4A. Reactions contained either no L30 or 44 pmol of wild-type or mutant L30 proteins. Results are expressed as means ± SEM.

We also analyzed several mutant L30 proteins for their ability to inhibit UGA recoding in our in vitro translation system. As shown in Figure 7, the L29A and M108A mutant proteins, which have wild-type levels of SECIS-binding activity, reduced UGA recoding by ~50%, similar to wild-type L30. However, the K36A and Y89A mutants, that are defective in SECIS-binding, only reduced UGA recoding by 9% and 4% respectively Thus, there was a correlation between the ability of L30 to bind to the SECIS and repress recoding in vitro.

### A naturally occurring SECIS mutation inhibits L30 binding

One interesting finding from the footprinting experiments was that L30 protected U40 in the SECIS core. A previous study identified a naturally occurring U to C point mutation at this position in the Selenoprotein N (SelN) SECIS element from a patient with a mild form of rigid spine muscular dystrophy [16]. SelN expression was impaired in this patient, and this mutation was shown to abrogate binding of SBP2 to the SelN and PHGPx SECIS elements [13,16]. We hypothesized that the mutant RNA may also be defective in the L30:SECIS interaction, as L30 and SBP2 have overlapping binding sites (Figure 2A). The ^32^P-labeled wild-type and U40C mutant SECIS RNAs were incubated with increasing amounts of purified L30 protein. The RNA:protein complexes were UV cross-linked, digested with RNase A, and analyzed by SDS-PAGE electrophoresis and autoradiography. As shown in Figure 2B, L30 cross-linked to the wild-type SECIS but not to the U40C mutant RNA. Thus, defects in selenoprotein synthesis in patients with mutant SECIS elements may not be solely due to an impaired SBP2:SECIS interaction.

## Discussion

As members of the L7Ae family, L30 and SBP2 share a similar RNA-binding domain. However, there are important differences in how the two proteins interact with the SECIS. Our RNA footprinting experiments revealed that the binding site of L30 centers on the SECIS core and internal loop. L30 protects nucleotides in the SECIS core, including the Gs in the two, sheared G•A tandem base pairs that form the putative kink-turn motif. The protein also protects many of the nucleotides in the internal loop. Thus, the L30 binding site described here overlaps with the regions that have been shown to be protected by SBP2 binding, including the SECIS core and 5’ side of the internal loop [14]. In addition to sharing these common nucleotide requirements, L30 and SBP2 each have unique determinants for binding to the SECIS. We show here that L30 protects the 3’ side of the internal loop, which is distinct from the known SBP2 binding site [14]. Likewise, SBP2 protects nucleotides in the upper part of helix 1, which are not part of the L30 binding site.

The fact that the binding sites of L30 and SBP2 overlap provides a molecular explanation for our earlier finding that the two proteins cannot interact simultaneously with the SECIS [9]. Our results also have implications for interpreting defects in SBP2:SECIS interactions that are associated with human disease. A naturally occurring point mutation in a highly conserved nucleotide in the SelN SECIS that was previously shown to disrupt SBP2 binding and selenoprotein synthesis also inhibited binding of L30. Thus, it is important to keep in mind that mutant SECIS elements may be defective in more than one function.

To date, the SECIS element is the only known cognate RNA for SBP2. In contrast, L30 binds to several other kink-turn containing targets, including the L30 pre-mRNA, L30 mature mRNA, and 28S ribosomal RNA. As the structure of the SBP2:SECIS complex has not been solved, we turned to the NMR and co-crystal structures of the yeast L30 protein in a complex with the L30 pre-mRNA to guide our mutational analysis. We chose to design site-specific mutations in regions defined as the RNA-binding interface for the L30:L30 pre-RNA interaction. Interestingly, the crystal structure of a 60S ribosomal subunit from the fresh water ciliate *Tetrahymena thermophila* was recently published, and it is the first such structure from an organism that synthesizes selenoproteins [4]. Using the published coordinates, we utilized several molecular modeling programs to analyze the structure of L30 on the ribosome where it is bound to helix 58 of the 26S rRNA (unpublished observations). *T. Thermophila* L30 appears to use a similar RNA-binding interface to bind to a kink-turn in helix 58, as the yeast protein uses to bind to the L30 pre-mRNA. Thus, we expected that some of the same amino acids might also be important for the L30:SECIS interaction. Indeed, the Y89A mutant protein was defective in binding to both the SECIS and the L30 RNA, its canonical target. This residue is equivalent to F85 in the yeast protein, where it is the most prominent amino acid contact in the L30:L30 pre-mRNA complex [29,30]. Based on mutagenesis studies, an aromatic group is required at this position for binding of the yeast protein to the L30 RNA [29], and a similar requirement may be true for the L30:SECIS interaction.

Unlike Y89, mutagenesis of K36 and K87 had selective effects on the interaction of L30 with the two target RNAs. The K36A mutation had a greater inhibitory effect on binding of the protein to the L30 RNA than to the SECIS. The equivalent amino acid in the yeast protein, K32, did not directly contact the RNA in the NMR and crystal structures of the L30:L30 RNA complex [30]. However, it was proposed that this highly conserved amino acid could play an important role by neutralizing the negatively charged phosphate backbone [29,30]. We also found that mutagenesis of L35, which is also in the α2 helical region, modestly reduced binding inhibition to both targets. This result was only statistically significant for the L30:L30 RNA interaction, however.

In contrast, we found that K87 is important for the L30:SECIS interaction, but is dispensable for binding to the L30 RNA. This was a particularly interesting result as K87 is part of a signature motif that has been identified for L30 [37]. It is well established that each L7Ae family member has a unique RNA-binding specificity. However the basis for this selectivity has not been well understood as the proteins share a relatively conserved RNA-binding domain. An elegant study by Gagnon et al. recently identified five signature amino acids flanking this region that are unique to each family member [37]. One amino acid is N-terminal to the RNA-binding domain whereas the other four residues comprise a motif that is C-terminal to the domain. The C-terminal motif in particular is quite different between family members, with respect to the structure and chemical properties of the conserved amino acids. Functional evidence was provided for the L7Ae and 15.5 kDa proteins, showing that the unique conserved amino acids are necessary although not sufficient to mediate specific binding to the appropriate cognate RNA. The consensus N-terminal amino acid for L30 and SBP2 is a basic residue, K26 in rat L30 and R665 in rat SBP2 (Figure 5A). K87, which is important for the L30:SECIS interaction, is the first amino acid in KYYR, the C-terminal signature motif for L30 [37]. This residue is also highly conserved in SBP2 (K733 in rat SBP2, see Figure 5), where it is part of this protein’s signature motif KAVP [37]. Interestingly, K is not present in the first position of the signature motifs for other eukaryotic L7Ae family members, including L7Ae, 15.5 kDa protein, rpL7a, Nhu2p, and Rpp38p [37]. The unique signature motifs that have evolved in L30 and SBP2 may explain the ability of these proteins to bind to the SECIS, which contains a large internal loop and a non-canonical kink-turn motif. As discussed above, the binding of L30 to the SECIS and the L30 RNA depends on Y89, which is the third amino acid in the KYYR motif. SBP2 has a V at this position, and mutational analysis of human SBP2 showed that this amino acid is important for the SBP2:SECIS interaction [38]. The fact that the signature motifs for L30 and SBP2 are not identical is consistent with the unique RNA-binding specificities of the two proteins, as L30 has been shown to bind to multiple cognate RNAs whereas the only known target for SBP2 is the SECIS element. An important future direction will be to solve the structures of the L30:SECIS and SBP2:SECIS complexes.

## Conclusions

The study presented here provides new insight into how ribosomal protein L30 recognizes the SECIS element. Our findings suggest that there are subtle differences in how L30 interacts with its different cognate RNAs. The results expand our knowledge of how L7Ae family members recognize different RNA targets.

## Methods

### Constructs

The wild-type rat PHGPx SECIS and U40C mutant constructs were previously described [13]. The rat PHGPx SECIS elements of 129 nucleotides and 102 nucleotides, which were used for RNA footprinting and REMSA analysis respectively, are described in [39]. The stem-loop L30 pre-mRNA was generated by annealing primers (Additional file 1: Table S1) in 10 mM NaCl and slow cooling the sample from 95°C to room temperature. The luc/UGA^258^/PHGPx and luc/UGA^258^/TR1 constructs were described in [40]. The L30 cDNA was amplified from rat liver cDNA (BD Bioscience) by polymerase chain reaction (PCR) and the products cloned into the Champion™ pET200 Directional TOPO® vector (Life Technologies) downstream of an N-terminal His_6_ tag. Mutants of L30 were constructed using the QuikChange Site-directed mutagenesis method (Stratagene), using the appropriate mutagenic primers (Additional file 1: Table S1). All constructs were verified by Sanger sequencing.

### RNA synthesis

Plasmid DNAs were linearized and used as templates for in vitro transcription. For UV cross-linking and REMSA analyses, RNAs were synthesized in the presence of [α^32^P] UTP (800 Ci/mmole; Perkin Elmer Easy Tides) using the RiboMAX™ Large Scale RNA Production Systems (T7) (Promega). Transcripts were purified using organic extraction followed by gel filtration using Micro Bio-Spin 30 Columns (BioRad). L30 RNA was gel purified and resuspended in 10 mM Tris HCl, pH 7.5. Unlabeled RNAs were prepared using the AmpliScribe T7-Flash Transcription kit (Epicentre) and purified as described above. For RNase footprinting experiments, cold synthetic transcripts were dephosphorylated with SuperSAP (Affymetrix), purified, and resuspended in nuclease-free water. Dephosphorylated transcripts were end-labeled in the presence of [γ^32^P] ATP (3000 Ci/mmole; Perkin Elmer Easy Tides) and T4 PNK (NEB) at 20 units/pmole RNA. The transcripts were gel purified on 8% acrylamide (19:1)/7M urea gels and eluted in 10 mM Tris HCl, pH 7.5, 1 mM EDTA, pH 8, 300 mM NaAc, pH 5.5 at 4°C overnight. Purified RNA was stored in 10 mM Tris HCl, pH 7.5 at -20°C.

### Protein purification

Recombinant rat L30 was expressed in BL21 Star (DE3) cells (Invitrogen). IPTG was added to 1 mM and the culture was grown at 37°C for 3 hr. The cells were harvested at 4°C, and the pellets washed with 1X PBS and frozen on dry ice. Purification buffers (PB) contained 20 mM HEPES-HCl, pH 8.0 and the indicated amounts of NaCl and imidazole. The frozen cell pellet was re-suspended in 20 ml of PB/0.2 M NaCl/20 mM imidazole, to which 60 kU rlysozyme (Novagen), 500 U of Benzonase Nuclease (Novagen) and two Complete, Mini, EDTA-free Protease Inhibitor Cocktail tablets (Roche) were added. The cells were further lysed by sonication on ice. The insoluble material was removed by two centrifugations of the lysate at ~16,000 × g for 15 min at 4°C. The cell lysate was filtered through a 0.45 μM filter and passed across a HisTrap TM FF crude column (GE Healthcare) using an AKTA Purifier (UPC-900) (GE Healthcare). The resin was sequentially washed with PB/0.2 M NaCl/20 mM imidazole, PB/1 M NaCl/20 mM imidizole, and PB/0.2M NaCl/40 mM imidazole. The protein was eluted with PB/0.2 M NaCl/250 mM imidazole and collected in 1 or 2 ml fractions. Fractions containing the pure protein were combined and dialyzed against 1 L of PB/0.2 M NaCl buffer for 2 hr at 4°C, which was then repeated with fresh buffer for an additional 2 hr at 4°C. The protein was dialyzed against PB/0.2 M NaCl containing 50% glycerol for 18 hr at 4°C. The dialyzed protein was aliquoted and stored at −70°C. Recombinant SBP2-CT was purified as previously described [40].

### RNase footprinting

End-labeled ^32^P-labeled PHGPx SECIS RNA was heated to 95°C and slow cooled to room temperature. The RNA (2.5 nM) was incubated in L30 binding buffer (30 mM Tris HCl, pH 8.0, 75 mM KCl, 5 mM MgCl_2_, 1 mM DTT, 0.04 μg/μL BSA (NEB), 10% glycerol, and 50 ng/μL yeast tRNA) with or without rat L30 protein (0.75 or 1.5 μM as indicated) at 30°C for 15 min. Reactions were cooled to room temperature over a 2 min period and then placed at 22°C for 2-5 min. The indicated amounts of RNase T1, A, or V1 (Ambion) were added to the appropriate samples and incubated at 22°C for 5 min. Enzymatic reactions were quenched with 20 μL Inactivation/Precipitation buffer (Ambion) and purified according to manufacturer’s directions. Samples were resuspended in 10 μL of dye-less loading buffer (95% formamide, 18 mM EDTA, and 0.025% SDS), heat-denatured at 95°C for 5 min, and separated in a denaturing 8% (19:1) polyacrylamide/7 M urea gel. The dried gels were visualized with a phosphorimager or on film.

Sequencing ladders were prepared by incubating end-labeled ^32^P-labeled PHGPx SECIS RNA (2.5 nM) in 1X Sequencing Buffer (Ambion) supplemented with 50 ng/μL yeast tRNA. The RNAs were incubated at 50°C for 5 min, cooled to 22°C and the indicated amounts of RNase T1, A, or V1 added. The samples were incubated, quenched, and purified as described above. Alkali ladders were prepared by incubating end-labeled ^32^P-labeled PHGPx SECIS RNA (2.5 nM) in 100 mM NaOH, 2 mM EDTA, pH 8.0, and 2 μg/μL yeast tRNA at 37°C for 3 min, to which 0.2 M Tris HCl, pH 8.0 (final) was added. The samples were frozen on dry ice and combined with an equal volume of denaturing loading buffer (10 M urea, 1X TBE).

### UV cross-linking

The ^32^P-labeled PHGPx SECIS or U40C mutant RNAs (10 fmol) were incubated in buffer containing 0.7X PBS, 11 mM DTT, 250 ng/μL yeast tRNA and RNAguard (Amersham). The indicated amounts of L30 were added last and the reactions incubated at 37°C for 30 min. The samples were irradiated on ice at 254 nm for 10 min in Costar 96-well polystyrene plates (Corning, Inc) using a Bio-Rad GS Genelinker. The RNA was digested with 20 U of RNase A (Fermentas) at 37°C for 1 hr. The samples were separated on a 15% SDS–PAGE (37.5:1) gel. The gels were dried and visualized using a phosphorimager.

### UGA recoding assays

*In vitro* transcribed RNAs (100 ng) were added to an *in vitro* translation reaction (25 μL) containing rabbit reticulocyte lysate (Promega), complete amino acid mix (Promega), Protector RNase Inhibitor (Roche), and 70 nM purified recombinant SBP2-CT protein. Purified recombinant L30 protein was added as indicated. Reactions were incubated at 30°C for 30 min and placed on ice for 15 min. Aliquots of the translation products (2.5 μL) were then added to the luciferase substrate (50 μL) in a well of a Microlite 1 microtiter plate (Thermoscientific). Luminescence was measured in 10 sec intervals using a Victor^3^ Multilabel Counter (Perkin Elmer).

### RNA Electrophoretic Mobility Shift Assays (REMSA)

The ^32^P-labeled SECIS RNA (10 fmol) was incubated in L30 binding buffer (30 mM Tris HCl, pH 8.0, 75 mM KCl, 5 mM MgCl_2_, 1 mM DTT, 0.04 μg/μL BSA (NEB), 10% glycerol, 50 ng/μL yeast tRNA, and 0.2 U/μL Protector RNase Inhibitor (Roche)) with the indicated final concentration of L30 protein at 30°C for 15 min. Samples were transferred to ice and then separated in non-denaturing polyacrylamide gels, either 6% (19:1) or 8% (29:1), in 0.5X TBE gel at 4°C. The dried gels were visualized with a Phosphorimager or on film. The ^32^P-labeled yeast L30 RNA was incubated in 350 mM KCl, 30 mM Tris HCl, 8.0, and 10 mM DTT at 60°C and allowed to slow cool to room temperature as previously described [41]. The binding reactions were then set-up and processed as described above. The reactions were separated in a non-denaturing 8% (29:1) polyacrylamide/0.5X TBE gel at 4°C.

### Statistical analysis

Data were analyzed using GraphPad Prism software. The results are expressed as means +/- standard error of the mean (S.E.M). Statistical significance is indicated as * (P < 0.05), ** (P < 0.01), or *** (P < 0.001).

## Abbreviations

PHGPx: Phospholipid Hydroperoxide Glutathione Peroxidase; REMSA: RNA Electrophoretic Mobility Shift Assay; RRL: Rabbit reticulocyte lysate; SBP2: SECIS-binding protein 2; Sec: Selenocysteine; SECIS: Selenocysteine insertion sequence; SelN: Selenoprotein N; TR1: Thioredoxin Reductase 1.

## Competing interests

The authors declare they have no competing interests.

## Authors’ contributions

AB, TF, and DD conceived and designed the experiments, and analyzed data. AB, TF, and TA performed experiments. AB and DD wrote the manuscript. All authors read and approved the final manuscript.

## Supplementary Material

Additional file 1: Table S1 Oligonucleotides used in this study. Click here for file
