# Supplementary material for: Identification of nucleotides and amino acids that mediate the interaction between ribosomal protein L30 and the SECIS element
Source: BMC Mol Biol. 2013 Jun 19;14:12. doi: 10.1186/1471-2199-14-12 (PMC3706390; doi:10.1186/1471-2199-14-12)
Supplement: Additional file 1: Table S1 — Oligonucleotides used in this study. [file 1471-2199-14-12-S1.doc]

Supplementary Table S1: Oligonucleotides used in this study.

| Primer Name | Primer Sequence (5’ to 3’) |
| --- | --- |
| L30 RNA Fwd | TAATACGACTCACTATAGGGACCGGAGTGTCGCAAGACGCAGAGATGGTCC |
| L30 RNA Rev | GGACCATCTCTGCGTCTTGCGACACTCCGGTCCCTATAGTGAGTCGTATTA |
| L30 L29A Fwd | AGTGGAAAGTACGTGCTGGGGTACAAACAGACT |
| L30 L29A Rev | AGTCTGTTTGTACCCCAGCACGTACTTTCCACT |
| L30 L35A Fwd | GGGTACAAACAGACTCTGAAGATGATCAGACAA |
| L30 L35A Rev | TTGTCTGATCATCTTCAGAGTCTGTTTGTACCC |
| L30 K36A Fwd | TACAAACAGACTCTGAAGATGATCAGACAAGGC |
| L30 K36A Rev | GCCTTGTCTGATCATCTTCAGAGTCTGTTTGTA |
| L30 K87A Fwd | GGCACAGCGTGTGGAAAATACTACAGAGTATGC |
| L30 K87A Rev | GCATACTCTGTAGTATTTTCCACACGCTGTGCC |
| L30 Y89A Fwd | GCGTGTGGAAAATACTACAGAGTATGCACACTG |
| L30 Y89A Rev | CAGTGTGCATACTCTGTAGTATTTTCCACACGC |
| L30 V91A Fwd | GGAAAATACTACAGAGTATGCACACTGGCTATC |
| L30 V91A Rev | GATAGCCAGTGTGCATACTCTGTAGTATTTTCC |
| L30 M108A Fwd | GATATTATTAGAAGCATGCCAGAACAGACTGGT |
| L30 M108A Rev | ACCAGTCTGTTCTGGCATGCTTCTAATAATATC |
| L30 E110A Fwd | ATTAGAAGCATGCCAGAACAGACTGGTGAGAAG |
| L30 E110A Rev | CTTCTCACCAGTCTGTTCTGGCATGCTTCTAAT |
